# A novel *KCND3* variant in the N‐terminus impairs the ionic current of Kv4.3 and is associated with SCA19/22

**DOI:** 10.1111/jcmm.70039

**Published:** 2024-08-24

**Authors:** Marlen Colleen Reis, Laura Mandler, Jun‐Suk Kang, Dominik Oliver, Christian Halaszovich, Dagmar Nolte

**Affiliations:** ^1^ Institute of Human Genetics Justus‐Liebig‐University Giessen Giessen Germany; ^2^ Department of Neurology Goethe‐University Frankfurt Frankfurt Germany; ^3^ Institute of Physiology Philipps‐University Marburg Marburg Germany; ^4^ Present address: Department of Neurology Justus‐Liebig‐University Giessen Giessen Germany; ^5^ Present address: Neuropraxis Frankfurt Germany

**Keywords:** ataxia, electrophysiology, *KCND3*, Kv4.3, movement disorder, neuropathology, SCA19/22

## Abstract

Spinocerebellar ataxias (SCAs) are a genetically heterogeneous group of autosomal dominant movement disorders. Among the SCAs associated with impaired ion channel function, SCA19/22 is caused by pathogenic variants in *KCND3*, which encodes the voltage‐gated potassium channel Kv4.3. SCA19/22 is clinically characterized by ataxia, dysarthria and oculomotor dysfunction in combination with other signs and symptoms, including mild cognitive impairment, peripheral neuropathy and pyramidal signs. The known *KCND3* pathogenic variants are localized either in the transmembrane segments, the connecting loops, or the C‐terminal region of Kv4.3. We have identified a novel pathogenic variant, c.455A>G (p.D152G), localized in the N‐terminus of Kv4.3. It is located in the immediate neighbourhood of the T1 domain, which is responsible for multimerization with the β‐subunit KChIP2b and thus for the formation of functional heterooctamers. Electrophysiological studies showed that p.D152G does not affect channel gating, but reduces the ionic current in Kv4.3, even though the variant is not located in the transmembrane domains. Impaired channel trafficking to the plasma membrane may contribute to this effect. In a patient with a clinical picture corresponding to SCA19/22, p.D152G is the first pathogenic variant in the N‐terminus of Kv4.3 to be described to date with an effect on ion channel activity.

## 1 INTRODUCTION

Spinocerebellar ataxias (SCAs) comprise a steadily growing, clinically and genetically heterogeneous group of neurodegenerative disorders that are inherited in an autosomal dominant manner. SCAs are clinically characterized by a combination of gait or stance ataxia, dysarthria, and oculomotor dysfunction. A wide spectrum of additional signs and symptoms such as tremor, dystonia, retinopathy, myoclonus, and altered nerve conductance can also occur. Neuropsychiatric features, such as depression, and intellectual decline are also common in ataxia patients. However, only in some cases the SCA subtype can be inferred from the symptoms observed. To date, 50 SCA loci have been reported that are associated with 40 disease genes.^1^, ^2^

Common genetic alterations underlying disease are repeat expansions in the coding or non‐coding sequence in the respective genes. Furthermore, conventional mutations or, more rarely, large deletions and duplications are found. Conventional mutations include point mutations in the coding sequence and at splice boundaries, as well as small deletions/insertions leading to frameshifts.^3^

The genes mutated in the rare SCA types have different functions in cell physiology. Among them are two that code for potassium channels. These include *KCNC3* (SCA13, OMIM #605259)^4^ and *KCND3*, which is associated with SCA19/22 (OMIM #607346).^5^ Pathogenic missense variants in *KCND3* associated with a neurological phenotype lead to a loss‐of‐function of the encoded voltage‐gated potassium channel Kv4.3.^6^, ^7^, ^8^, ^9^, ^10^, ^11^, ^12^, ^13^, ^14^, ^15^, ^16^, ^17^

The large family of voltage‐gated potassium channels includes the Kv4 subgroup (*shal* family), which consists of three channels, Kv4.1–Kv4.3. The Kv4 channels are found in both neurons and heart muscle cells and are involved in regulating various physiological processes such as neuronal excitability and muscle contractions.^18^ Kv4.3 subunits share the common architecture of all Kv channels, composed of six transmembrane domains (S1‐S6), a pore loop that controls the inactivation process, and cytoplasmic C‐ and N‐termini.^19^ At the N‐terminus, the T1 domain, which comprises amino acids (aa) 40–148, functions as an assembly domain for building the pore‐forming tetramer.^20^ The T1 domain also binds β‐subunits, the Kv channel interacting proteins (KChIPs). KChIP2 binds to Kv4.3 in heart and brain and modulates channel activity.^21^

Kv4.3 is highly expressed in the cerebellum,^22^ where it produces an A‐type K^+^‐current that impacts excitability and action potentials of Purkinje cells, granule cells and interneurons. In addition, Kv4.3 is responsible for the transient outward K^+^‐current (I_to_) in cardiomyocytes, which is important for the early repolarization phase of the cardiac action potential.^23^ Gain‐of‐function variants of Kv4.3 lead to a cardiac phenotype, including Brugada syndrome^24^ and sudden unexplained death syndrome.^25^

In this study, a novel likely pathogenic *KCND3* variant in the N‐terminus of Kv4.3 is functionally characterized to elucidate its effects on channel gating and its association to SCA19/22.

## 2 PATIENTS AND METHODS

### 2.1 Patients

The index patient was diagnosed with an ataxic movement disorder in a hospital specialized in movement disorders. Informed consent to donate blood for genetic testing in accordance with the guidelines of the German Genetic Diagnostics Act was given by the patient. The study was performed in accordance with the principles of the Declaration of Helsinki and was approved by the ethics committee of the Justus‐Liebig‐University of Giessen (AZ24/14_KCND3).

### 2.2 Genetic Analysis

Genomic DNA was extracted from peripheral blood samples using standard procedures. Repeat length expansions at loci SCA 1–3, 6–8, 10, 12 and 17 and pathogenic variants at loci SCA 13, 14, 23, 27, 28, and 35 have been previously excluded. DNA was analysed for variants in *KCND3* (ENSG00000171385) by amplification and sequencing of the coding exons and the flanking sequence of introns in transcripts ENST00000369697 (six coding exons) and ENST00000315987 (seven coding exons). Primer sequences are given in Table S1A.

Analysis was supplemented by whole exome sequencing. SureSelect XT HS Human All Exon V8 kit (Agilent, Santa Clara, CA, United States) was used for enrichment. The prepared library was checked with a Qubit 3.0 fluorometer (ThermoFisher Scientific, Waltham, MA, United States). Sequencing was performed on an Illumina NovaSeq platform (Illumina, San Diego, CA, United States). The mean coverage of exons was at least >20× with >99.3%, and >50× with 95.5% targeted bases covered. GRCh37 was used as the reference genome. Filter criteria for pathogenic variants were minor allele frequency (MAF) <0.01 in the gnomAD v2.1.1 database.^26^ An ataxia panel comprising 111 genes (Text S1B) was analysed prior to a whole exome. Varvis software v.1.23.3 (Limbus Medical Technologies GmbH, Rostock, Germany) was used for analysis.

### 2.3 *KCND3* and *KCNIP2* plasmids

The open reading frame (ORF) of wild‐type (WT) *KCND3* transcript variant 1 (NM_004980) was inserted in a pcDNA3.1+/C‐eGFP vector using enzymes HindIII (5′‐end) and NotI (3′‐end) to obtain plasmid pcDNA3.1‐*KCND3*(WT)‐C‐eGFP (*KCND3*‐WT). Expression results in a Kv4.3 fusion protein with an eGFP‐tag at its C‐terminus. Base change c.455A>G was introduced to obtain variant plasmid pcDNA3.1‐*KCND3*(p.D152G)‐C‐eGFP (*KCND3*‐p.D152G). In addition, the ORF of β‐subunit *KCNIP2* transcript variant 3 (NM_173192.2, codes for KChIP2b) was inserted in pcDNA3.1+/N‐mCherry using KpnI (5′‐end) and BamHI (3′‐end) sites to obtain plasmid pcDNA3.1‐mCherry‐N‐*KCNIP2* (*KCNIP2*). Plasmids were purchased at GenScript Biotech (Piscataway Township, New Jersey, USA). Competent JM109 *E.coli* cells (Promega GmbH, Madison USA) were transformed with 100 ng of delivered construct for subsequent midi plasmid preparation (Qiagen GmbH, Hilden, Germany). Integrity of the selected clones was confirmed by sequencing.

### 2.4 Cell culture and transfection

Chinese hamster ovary (CHO) dhFr^−^ cells (CRL 9096, ATCC) were cultured in Minimal essential medium (MEM) Alpha medium (Gibco Waltham, MA, United States), supplemented with 10% fetal calf serum (FCS) (Invitrogen, Waltham, MA, United States), 1% penicillin/streptomycin (Invitrogen). Cells were seeded on coverslip glasses and cultured at 37°C and 5% CO_2_. Cells were transfected with 3 μg *KCND3*‐WT or *KCND3*‐p.D152G, using JetPEI DNA transfection reagent according to manufacturer's instructions (Polyplus transfection, Illkirch‐Graffenstaden, France). For co‐expressions, 1.5 μg of *KCND3*‐WT or *KCND3*‐p.D152G and 1.5 μg of *KCNIP2* were transfected. Recordings were made 24–48 h after transfection.

### 2.5 Electrophysiological recordings

Whole‐cell recordings were performed with a EPC10 USB amplifier controlled by the PatchMaster Software (HEKA Elektronik GmbH, Reutlingen). The patch electrodes were pulled from 1.0 mm borosilicate capillary glass (Science Products GmbH, Hofheim) using a P2000 pipette puller (Sutter Instruments, Novato, CA). The electrode resistance was 1–3 MΩ for whole‐cell recording. Series resistance (R_S_) was between 1 and 20 MΩ.

The intracellular solution consisted of 135 mM KCl, 5 mM HEPES, 5 mM EGTA, 2.5 mM MgCl_2_, 2.41 mM CaCl_2_, 3 mM Na_2_ATP, 0.1 mM Na_3_GTP (pH 7.3). The bath solution consisted of 114 mM NaCl, 5.8 mM KCl, 0.9 mM MgCl_2_, 1.3 mM CaCl_2_, 10 mM HEPES, 5.6 mM glucose, 0.7 mM NaH_2_PO_4_ (pH 7.3).

### 2.6 Data analysis

To calculate conductance‐voltage relations, currents were evoked by applying 800 ms voltage steps starting at −70 mV up to potentials of +100 mV in 10 mV increments. The voltage dependent conductance (G) was obtained by fitting I/V‐relations with

$$\;I=G_{L}*V+G*\left(V-E_{K}\right)+I\_0$$

with

$$G=G_{\max}/\left(1+\exp\left(\frac{V_{\text{half}}-V}{s}\right)\right)$$

where G_L_ denotes the leak conductance; E_K_ the reversal potential for K^+^, which was calculated as −81 mV; G_max_ the maximal conductance; I_0 the background current; V_half_ the voltage at half maximal conductance, and s as the slope factor. Inactivation kinetics were determined by fitting the evoked currents at 70 mV from 0.132 to 0.863 s with:

$$y_{\text{inact}}=y_{0}+A\times \exp\left(-\frac{t-t_{0}}{\tau }\right).$$

with t_0_ = 0.1.

Steady‐state inactivation was determined as follows. From a holding potential of −75 mV, a 730 ms pre‐pulse was given to potentials between −100 and +50 mV in 10 mV increments, followed by a 250‐ms test pulse to +20 mV. Steady‐state inactivation curves were fitted using the Boltzmann sigmoidal equation:

$$I=I_{0}+I_{\max}/\left(1+\exp\left(\frac{V_{\text{half}\left(\text{inact}\right)}-V}{s\left(\text{inact}\right)}\right)\right)$$

To determine the time course of recovery from inactivation, an inactivating pre‐pulse was given by stepping from −100 to 20 mV for 2000 ms. This was followed by a step to −100 mV for varying durations, after which a test pulse was given (+40 mV for 350 ms). The initial duration was 5 ms and doubled for each repetition, up to a final value of 2.56 s. Current amplitude (I) values at 40 mV plotted against the interpulse interval and fitted with:

$$y_{\text{recov}}=y_{0}+A\times \exp\left(-\frac{t-t_{0}}{\tau }\right)$$

with t_0_ = 0 and normalized to maximum (I_max_).

Analysis and curve fitting of electrophysiological data were performed using Igor Pro 8 (WaveMetrics, Inc. Portland, USA). Statistical analysis was done using Igor Pro 8, Excel (Microsoft Office, Redmont, USA), or R Studio (The R Foundation for Statistical Computing, Vienna, Austria). Results are given in mean ± standard error of the mean (S.E.M.) and were compared with two‐sided unpaired t‐tests. The slope factor (inact) was tested with Wilcoxon Rank test. Since log(τ) is normally distributed, the time constant was also tested with t‐tests.

### 2.7 Confocal Imaging

Confocal imaging was performed after co‐transfection of CHO cells with 0.75 μg plasmids *KCND3*‐p.D152G, or *KCND3*‐WT in combination with 0.5 μg Lyn11‐mCFP.^27^ Lyn11‐mCFP was used as a membrane marker. Co‐transfection of Lyn11‐mCFP with *KCNIP2* and *KCND3*‐WT, or *KCNIP2* and *KCND3*‐p.D152G was performed at ratios of 2:3:3 (0.5 μg:0.75 μg:0.75 μg).

Confocal imaging was performed on an upright LSM 710 Axio Examiner microscope with a W‐Plan‐Apochromat 63× 1.0 VIS‐IR water immersion objective using ZEN2009 software (Carl Zeiss, Jena, Germany). Laser lines at 405 nm (diode laser) and 561 nm (diode pumped solid state laser) were used to excite CFP and mCherry. The wavelength ranges for detection were 463–556 nm and 578–696 nm, respectively. eGFP was excited with an argon laser at 488 nm and fluorescence emission was recorded at 493–575 nm. The images were prepared and the fluorescence intensity profiles were analysed using Fiji.^28^

## 3 RESULTS

### 3.1 Clinical findings

According to the medical history, the patient was addicted to heroin from the age of 19 to 21. This was followed by many years of severe alcohol abuse up to the age of 53, which led to a hepatocellular carcinoma with subsequent liver surgery. Restless leg syndrome was diagnosed at the age of 49. The patient presented to a specialized outpatient clinic for movement disorders for the first time at the age of 54. Ataxic gait instability with a tendency to fall was observed. The knee–heel trial was ataxic, a tightrope walk was only possible with support for a short time. The Romberg test was also positive. Dysarthria and dysphagia were observed. There was gaze nystagmus to the right and left, double vision was denied. Mild intention tremor was noted in the finger‐nose test. The biceps and triceps tendon reflexes and the radial periosteal reflex were weakly preserved on both sides. A stocking‐like hypesthesia was noted in the middle of the lower legs. Cognitive abilities were preserved. These findings correspond to a Scale for the Assessment and Rating of Ataxia (SARA) score^29^ of 7. At age 54, cMRI showed no further abnormalities except for microangiographic changes in the spinal cord, basal ganglia and pons. The patient deceased at age 58.

**FIGURE 1:**
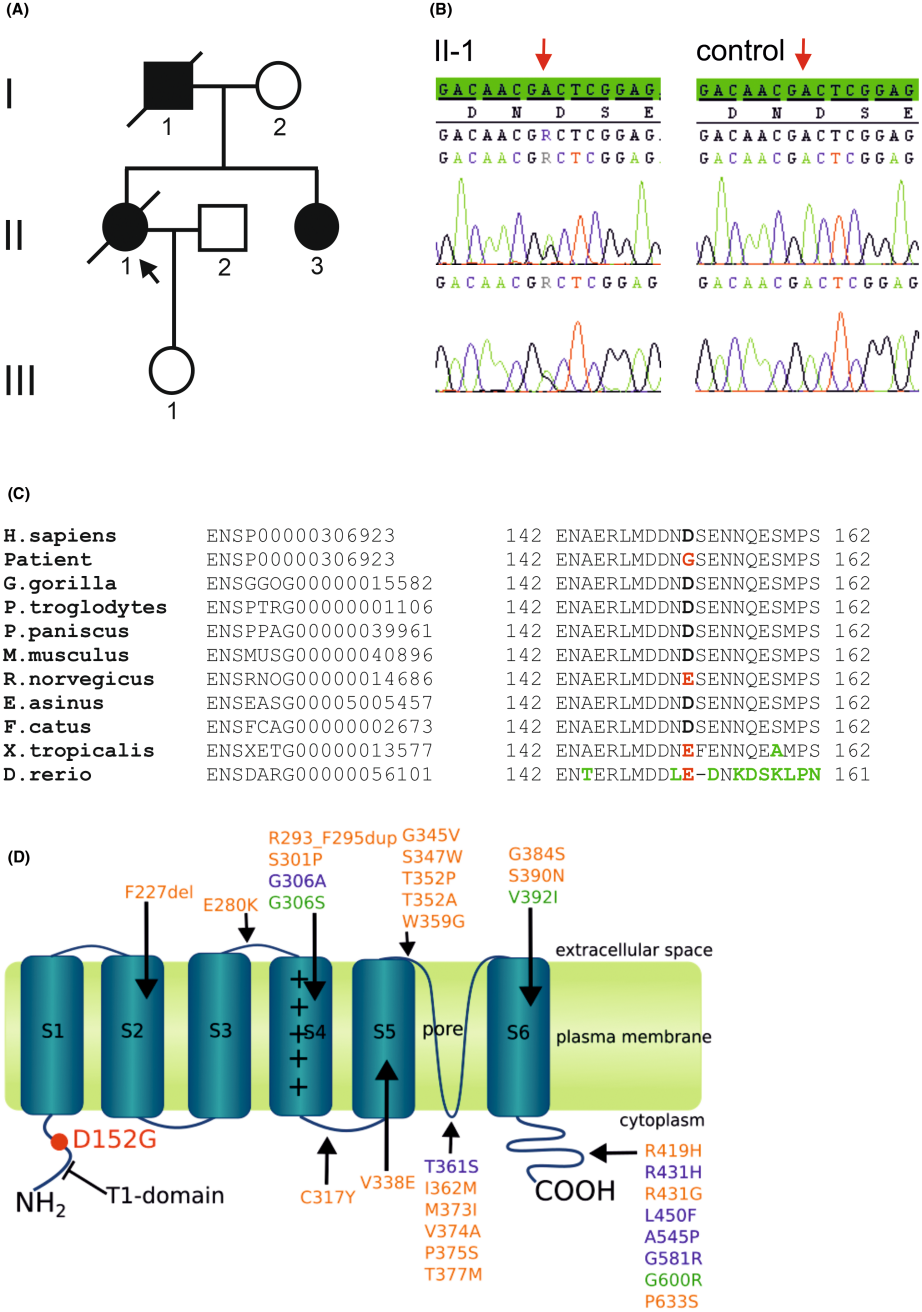
(A) Pedigree of the family of the index patient. Black symbols indicate affected probands. The index patient is marked by an arrow. (B) Electropherograms of *KCND3* sequences of the index patient and a control. The A>G transition at c.455 is indicated by an arrow. (C) Amino acid sequence alignments Kv4.3 orthologs. Name of species are given on the left. Amino acid mutated in the index patient is evolutionarily highly conserved and is highlighted in red. Non‐conserved amino acid residues are shown in green. H., *Homo*; G., *Gorilla*; P., *Pan*; M., *Mus*; R., *Rattus*; E.; *Equus*; F., *Felis*; X., *Xenopus*; D., *Danio*. (D) Schematic illustration of Kv4.3. S1 to S6 represent the transmembrane domains, while S4 is the voltage sensor. Amino acid changes that are associated with SCA19/22 are shown in orange. Changes that are associated with a cardiac phenotype (Brugada syndrome, sudden unexplained death syndrome, early repolarization syndrome, atrial fibrillation) are indicated in blue. Green indicates variants found in both patients with SCA19/22 and those with cardiac disease, or in patients with combined symptoms. The novel amino acid change p.D152G is located in the N‐terminus and is marked in red.

Initially, the patient was thought to have a slowly progressive cerebellar syndrome with cerebellar atrophy as a result of long‐term alcohol abuse. However, a thorough survey of the family history revealed that the index patient's father and older sister suffered from a similar movement disorder (Figure 1A). The family history therefore indicated a genetic cause with an autosomal dominant inheritance.

### 3.2 Genetic Findings

Repeat length of loci SCA 1–3, 6–8, 10, 12 and 17 were in the normal range in the index patient (II‐1, Figure 1A). At loci SCA 13/14, 23, 27, 28, and 35 no pathogenic variants were present. Sanger sequencing of coding exons and flanking intronic regions of *KCND3* (loci SCA19/22) revealed a heterozygous variant in exon 1 of transcript ENST00000315987. The variant, an adenosine (A) to guanine (G) transition at position 455 (c.455A>G) (Figure 1B) leads to an aa change replacing aspartic acid by glycine at position 152 (p.D152G) of Kv4.3. The variant is not present in databases such as Ensembl (https://www.ensembl.org), but is mentioned twice (2/1,461,878) in the gnomAD genome browser (https://gnomad.broadinstitute.org/) without further information on the probands. *In silico* analysis using MutationTaster2^30^ predicts the variant c.455A>G to be disease causing, whereas Polyphen2^31^ estimates a benign variant. However, aspartic acid at position 152 is strongly conserved in primates and rodents, with the exception of the rat (Figure 1C).

Whole‐exome sequencing was performed to determine whether the patient had other potentially pathogenic variants that could explain her movement disorder. The use of a SCA panel comprising 111 genes again revealed the variant c.455A>G in *KCND3*, which was already detected by Sanger sequencing. Bioinformatic tools for exome data, such as MetaSV^32^ and MetaLR,^33^ predicted the change as damaging with scores of 0.88149 and 0.90676, respectively. Prior to the functional analyses, the c.455A>G variant was classified as class 4/ probably pathogenic according to the ACMG guidelines^34^ with criteria PM1, PM2, PP2, PP3 and PP4.

Additionally, a heterozygous variant in the *ATM* gene (NM_000051.4: c.7891G>A; p.A2631T) in which homozygous or compound heterozygous variants are associated with ataxia‐telangiectasia (OMIM #208900) was identified. Further analysis of a clinical exome and copy number variations revealed a deletion on chromosome 16 affecting the *carboxylesterase 1* gene (*CES1*, NM_001025194.2; OMIM *114835), which is involved in drug metabolism.^35^ However, these two pathogenic variants are not directly related to the ataxic movement disorder observed in the patient and will be discussed later.

Since the new variant p.D152G is only mentioned twice in genome databases, bioinformatic prediction programs predominantly assume a deleterious effect, and a conserved aa residue is affected, the variant was examined for its channel properties.

### 3.3 Effect of p.D152G on Kv4.3 channel activation

**FIGURE 2:**
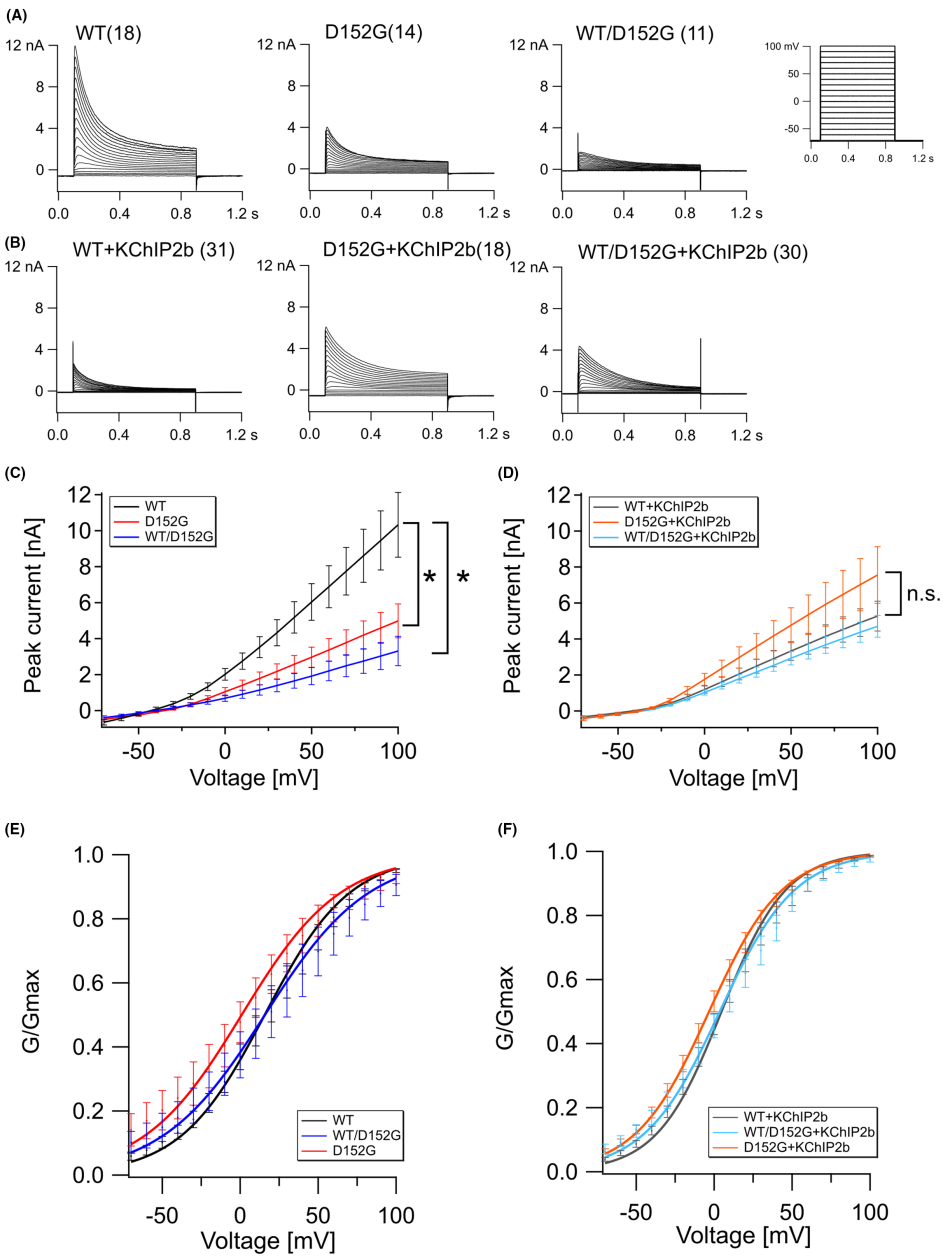
Analysis of Kv4.3 ion channel function as a homopolymer and as a heteropolymer with KChIP2b. (A) Current measurements of Kv4.3 WT, Kv4.3 p.D152G, and Kv4.3 WT/p.D152G (molar ratio 1:1). The number of independent experiments is given in parentheses. (B) Current measurements of Kv4.3 WT, Kv4.3 p.D152G, and Kv4.3 WT/p.D152G co‐expressed with KChIP2b (molar ratio 1:1). Number of independent experiments is given in parentheses. (C, D) Peak current amplitude of WT and variant channels in mean and S.E.M. Currents at 100 mV were tested with two‐tailed unpaired t‐test (*p < 0.05). (E) Normalized conductance (G/G_max_) of Kv4.3 channels alone, and (F) with KChIP2b.

The aa change p.D152G is located in the N‐terminus of Kv4.3 (Figure 1D) and is in close proximity to the T1 domain further N‐terminal, which extends from aa 40 to 148. T1 is responsible for the tetramerization of the channel and the binding of KChIP2b. Due to the proximity of p.D152G to the T1 domain, an effect on tetramerization and KChIP2b binding and therefore Kv4.3 function was hypothesized. To investigate the effect of p.D152G on Kv4.3 function and channel activity, WT and variant channels were analysed by whole‐cell patch‐clamp electrophysiology (Figure 2A,B). First, constructs encoding the WT Kv4.3 channel, variant (p.D152G) channel, or an equal mixture of WT and variant channel (WT/p.D152G) were expressed in CHO cells (Figure 2A). Strikingly, mutant Kv4.3 showed significantly less potassium current than the WT when expressed alone (*p* = 0.022; WT: 10.3 ± 1.8 nA vs p.D152G: 5 ± 0.9 nA at 100 mV; Figure 2C). Furthermore, co‐expression of WT and variant channel subunits, predicted to result in mixed heteromeric channel populations, showed significantly reduced current level as well (3.3 ± 0.8 nA; *p* = 0.015; Figure 2C). These results indicate that p.D152G impairs Kv4.3 function and suppress functional WT subunits in heterotetramers, most likely in a dominant‐negative manner.

Since Kv4.3 normally forms heterooctamers with the β‐subunit KChIP2 in brain and heart tissue, Kv4.3 constructs were co‐expressed with KChIP2b in equal parts (Figure 2B). It should be noted that 1.5 μg of Kv4.3 and 1.5 μg of KChIP2b are used per transfection instead of 3 μg of Kv4.3 in assays without KChIP2b. For this reason, the current amplitudes with and without KChIP2b cannot be compared directly. In these co‐expression experiments, WT, variant and heterotetramers showed similar current amplitude levels: WT + KChIP2b: 5.3 ± 0.8 nA; p.D152G + KChIP2b: 7.6 ± 1.5 nA; WT/p.D152G + KChIP2b: 4.7 ± 0.6 nA (Figure 2D). Overall, p.D152G has a negative effect on channel function and decreases the current amplitude. However, the negative effect is not detected by co‐expression with KChIP2b and formation of heterooctamers. One possibility is that co‐expressing KChIP2b might attenuate the negative effect, but the results are not significant (Figure 2D).

To assess if p.D152G influences the channel's voltage dependence, normalized conductance of WT and variant channels was fitted with a Boltzmann function, where V_half_ indicates the potential at half‐maximum activation, and the slope factor defines the steepness of voltage dependence (Figure 2E,F; Figure S1A,B). The V_half_‐value of variant channels (1 ± 9.7 mV) was less positive compared to Kv4.3 WT channels (15.8 ± 4.6 mV), but given the experimental variance, this shift was not significant. V_half_ of heteromeric WT/p.D152G channels (15.9 ± 9.9 mV) did not differ from Kv4.3 WT (Table 1).

**TABLE 1:** Gating parameters for Kv4.3 WT, variant p.D152G and heterotetramer WT/p.D152G, alone or co‐expressed with KChIP2b.

	WT	p.D152G	WT/p.D152G	With KChIP2b (1:1)		
				WT	p.D152G	WT/p.D152G
Activation (*n*)	18	14	11	31	18	30
V_half_ [mV]	15.8 ± 4.6	1 ± 9.7	15.89 ± 9.9	4.8 ± 3.4	−3.2 ± 2.9	3.62 ± 4.3
Slope [mV]	27.2 ± 2.1	31.7 ± 4.6	33.3 ± 6.3	20.8 ± 0.9^b^ ^,^ *	23.6 ± 0.9^a^	24.19 ± 1.9
Inactivation kinetics (*n*)	10	12	10	21	16	22
τ (70 mV) [ms]	160.2 ± 12.2	175.9 ± 21.8	177.8 ± 15.1	195.3 ± 19.6	161.8 ± 6.8	188.2 ± 8.7
Steady‐state inactivation (*n*)	16	10	19	27	18	33
V_half(inact)_ [mV]	−49.1 ± 2.8	−51.2 ± 2.6	−59.4 ± 3.2^a^	−42.9 ± 3	−38.2 ± 1.9^b^ ^,^ **	−40.1 ± 2.7^b^ ^,^ **
Slope (inact) [mV]	21.7 ± 4.2	17.1 ± 2.9	27.6 ± 2.1	16.8 ± 3.2	9.8 ± 2.4^b^ ^,^ *	17.5 ± 2.9^b^ ^,^ *
Recovery (*n*)	19	11	18	29	18	35
τ [ms]	137.4 ± 31.1	201.1 ± 41.8	190.7 ± 44.6	75.4 ± 9	80.4 ± 9.6	115 ± 19.5

*Note*: Numbers are given in mean ± S.E.M.

^a^ Significantly different to WT or WT + KChIP2b (*p* < 0.05).

^b^ Significantly different to channel without KChIP2b.

* *p* < 0.01.

** *p* < 0.001.

Co‐expression with KChIP2b resulted in a slight shift of V_half_ towards more negative voltages, which was similar for each channel variant (WT: 4.8 ± 3.4 mV; p.D152G: −3.2 ± 2.9 mV; WT/p.D152G: 3.6 ± 4.3 mV; Table 1). The voltage dependence (slope factor) of WT channels shifted significantly by co‐assembly with KChIP2b (*p* = 0.002; WT: 27.2 ± 2.1 mV; WT + KChIP2b: 20.8 ± 0.9 mV). For p.D152G and WT/p.D152G channels, this shift was not significant. However, the conductance curve of the WT channel + KChIP2b was steeper than the variant channel + KChIP2b (slope factor, *p* = 0.041; p.D152G + KChIP2b: 23.6 ± 0.9 mV).

### 3.4 Effect of p.D152G on Kv4.3 and KChIP2b inactivation voltage dependence and kinetics

**FIGURE 3:**
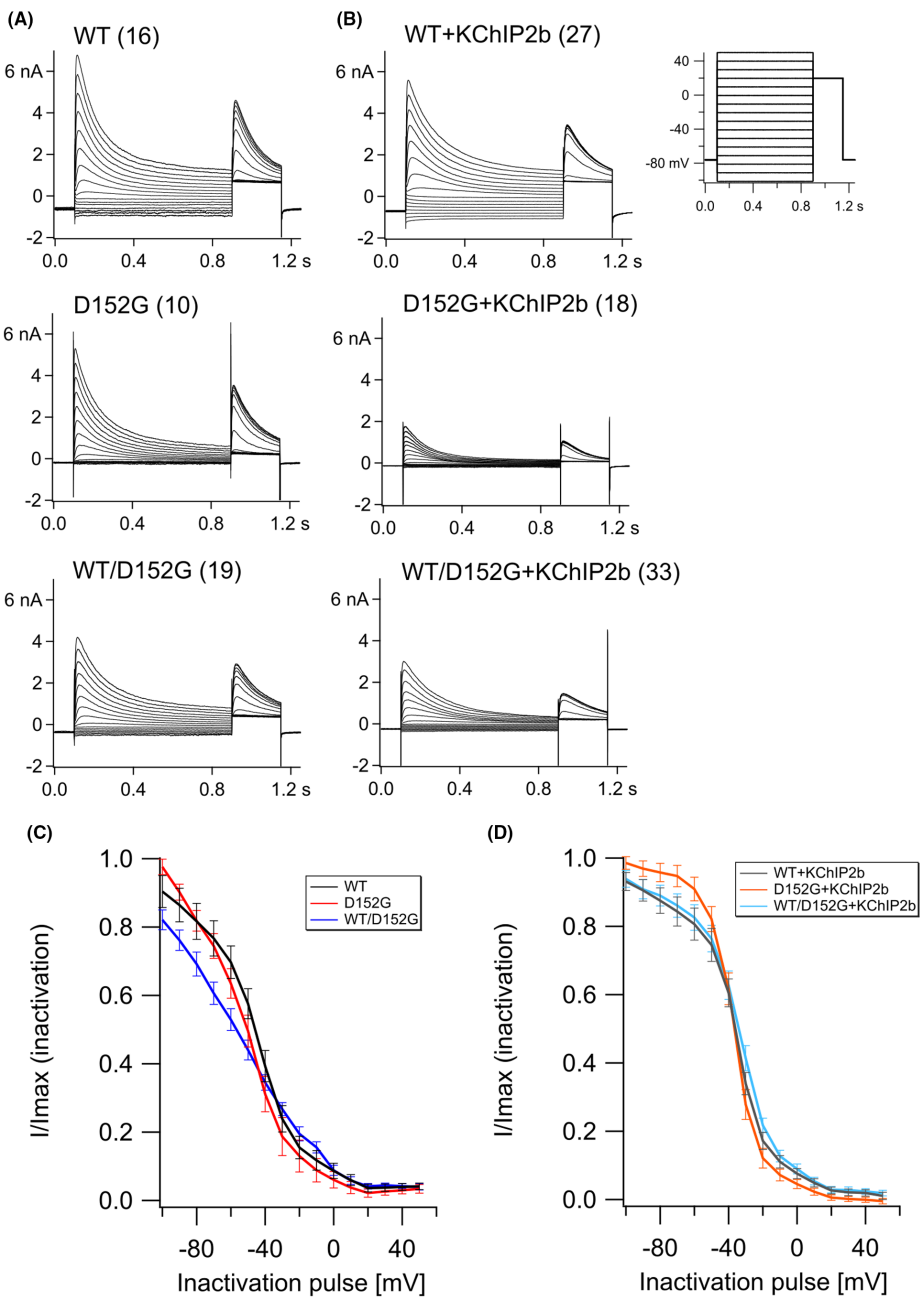
Inactivation kinetics of Kv4.3 with and without KChIP2b. (A) Current measurements of Kv4.3 WT, Kv4.3 p.D152G, and Kv4.3 WT/p.D152G (molar ratio 1:1). The number of independent experiments is given in parentheses. (B) Current measurements of Kv4.3 co‐expressed with KChIP2b (molar ratio 1:1). Number of independent experiments is given in parentheses. (C, D) Peak currents were normalized to I_max_ and leak current (I_0_) of their Boltzmann fits (I/I_max_) and plotted as a function of inactivation pulse voltage. (C) I/I_max_ of channels alone, and (D) with KChIP2b.

The inactivation behaviour of WT and variant channels were examined using a steady‐state inactivation protocol (Figure 3A,B; Figure S1C,D). V_half(inact)_ is the voltage at which 50% of the channels are inactivated after a given pre‐pulse. V_half(inact)_ values of the WT and variant channels were not different (Table 1), but interestingly, the heterotetramers of WT/p.D152G showed a significantly reduced V_half(inact)_ compared to the WT, meaning that the WT/p.D152G channels inactivate at a more negative potential than the WT (*p* = 0.03; WT: −49.1 ± 2.8 mV; WT/p.D152G: −59.4 ± 3.2; Table 1, Figure 3C). With co‐expressed KChIP2b, V_half(inact)_ did not differ between WT and variant (WT + KChIP2b vs p.D152G + KChIP2b; WT + KChIP2b vs WT/p.D152G + KChIP2b). However, V_half(inact)_ of variant channel alone versus variant channel +KChIP2b were significantly different (*p* = 0.0007; p.D152G: −51.2 ± 2.6 mV; p.D152G + KChIP2b: −38.2 ± 1.9 mV). Similarly, V_half(inact)_ of WT/p.D152G channels was more negative than WT/p.D152G + KChIP2b (*p* = 0.0001; WT/p.D152G + KChIP2b: −40.1 ± 3 mV). This shows that channels with KChIP2b inactivate at less negative potentials than without. Furthermore, the slope factor of the steady‐state inactivation curve was significantly different between p.D152G and p.D152G + KChIP2b (*p* = 0.0016; p.D152G: 17.1 ± 2.9 mV; p.D152G + KChIP2b: 9.8 ± 2.4 mV) and between WT/p.D152G and WT/p.D152G + KChIP2b (*p* = 0.0017; WT/p.D152G: 27.6 ± 2.1 mV; WT/p.D152G + KChIP2b: 17.5 ± 29 mV).

**FIGURE 4:**
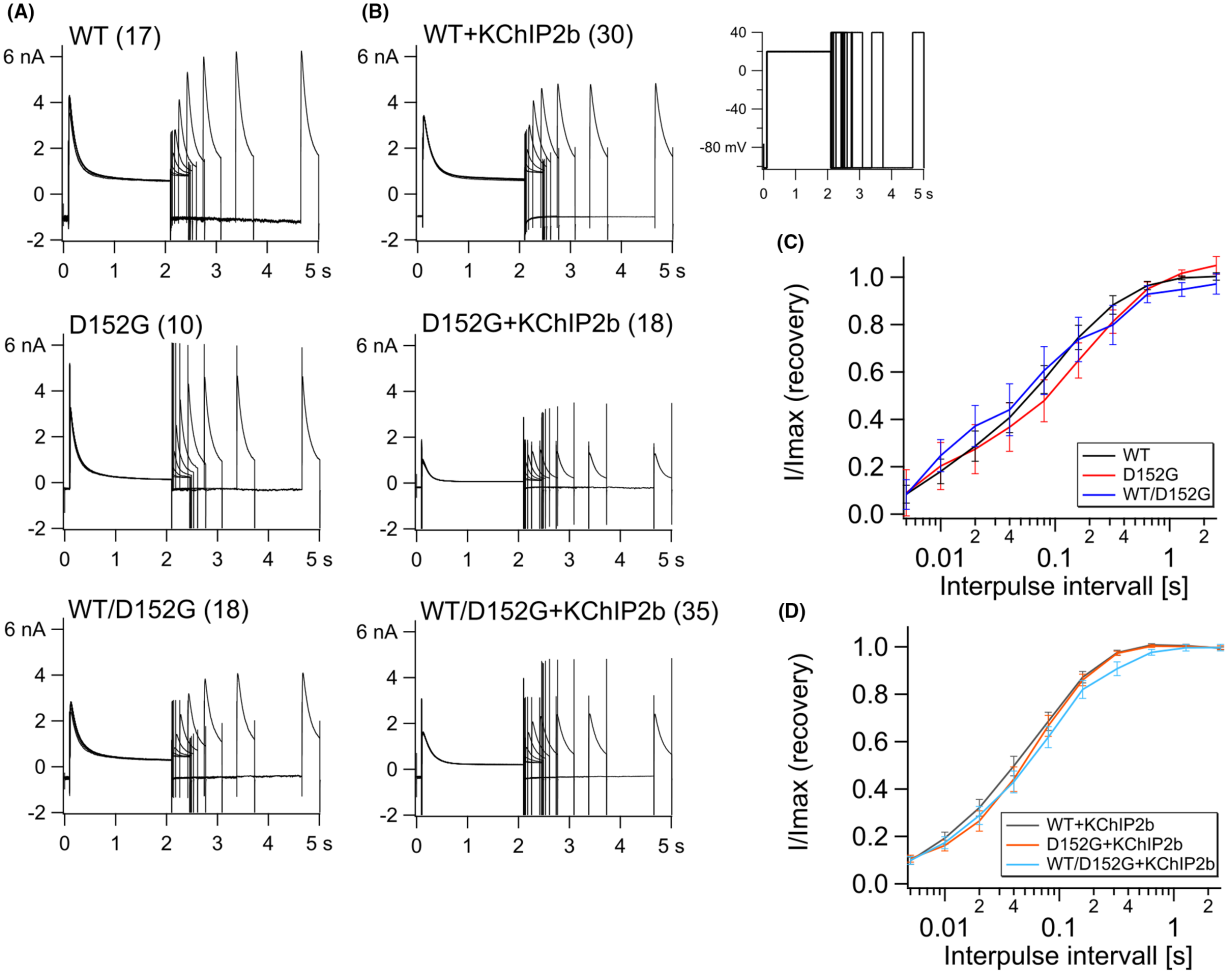
Recovery from inactivation kinetics of Kv4.3 with and without KChIP2b. (A) Current measurements of Kv4.3 WT, Kv4.3 p.D152G, and Kv4.3 WT/p.D152G (molar ratio 1:1). The number of independent experiments is given in parentheses. (B) Current measurements of Kv4.3 co‐expressed with KChIP2b (molar ratio 1:1). The number of independent experiments is given in parentheses. (C, D) Fits of peak current were normalized (I/I_max_) and plotted as a function of interpulse interval with logarithmic scale. (C) Fits of channels alone, and (D) with KChIP2b.

The time course of Kv4.3 inactivation during a given potential was determined by fitting the inactivation curve of the activation protocol at 70 mV with an exponential (Figure 2). Tau (τ) of WT, p.D152G and WT/p.D152G channels at 70 mV were not significantly different (Table 1; Figure S1E). Similarly, the time course for recovery from inactivation was not significantly different between WT and variant Kv4.3 (Figure 4A,B). Normalized current amplitudes for test pulses after different interpulse intervals showed similar curves for WT and variant channels (Figure 4C,D; Figure S1F).

### 3.5 Subcellular localization of Kv4.3 channels

To test whether the difference in channel activity between Kv4.3 WT and variant p.D152G was due to differences in subcellular localization, live‐cell imaging of transfected CHO cells was performed. Transfected Kv4.3 WT was partially located in the cytoplasm but showed a discernible concentration at the cell edge, indicating spatial plasma membrane localization (Figure S2A). Kv.4.3 p.D152G appeared to be less abundant at the cell membrane, with predominant localization rather in the cytoplasm (Figure S2B).

Co‐transfection of KChIP2b with Kv4.3 WT also results in distribution at the cell membrane and in the cytoplasm (Figure S2C). The fluorescence intensity profile shows that Kv4.3 WT/KChIP2b intensity peaks overlap at the cell edge, indicating localization as heterocomplexes at the cell membrane, as expected (Figure S2C.5). However, Kv4.3 p.D152G/KChIP2b complexes are also localized at the cell membrane (Figure S2D). Taken together, these results suggest that KChIP2b supports Kv4.3 localization to the cell membrane via heterocomplex formation.

In conclusion, p.D152G reduces ionic current in Kv4.3. Mislocalization of variant channels may contribute to this effect. As a heterooctamer together with KChIP2b, Kv4.3 may be more effectively transported to the cell membrane. In electrophysiological recordings, KChIP2b causes WT/ variant channels to open at more negative voltages and inactivate at less negative voltages.

## 4 DISCUSSION

In a family with a progressive ataxic movement disorder, three pathogenic variants were detected in the index patient by applying a 111‐gene ataxia panel, a whole exome and search for copy number variations. These variants are the loss of one copy of the *CES1* gene on chromosome 16, a heterozygous base change in *ATM* (c.7891G>A; p.A2631T), and another heterozygous pathogenic variant in *KCND3* (c.455G>A; p.D152G). It was initially assumed that the patient had a slowly progressive cerebellar syndrome as a result of many years of alcohol and drug abuse. The detected deletion of one copy of the *CES1* genes on the chromosome 16, which is associated with drug metabolism,^35^, ^36^ could represent a genetic component of the patient's addiction problem. However, the *CES1* gene deletion does not explain the movement disorder observed in the family. The heterozygous variant c.7891G>A (p.A2631T) was detected in *ATM*. Homozygous or compound heterozygous variants in *ATM* are associated with ataxia‐telangiectasia, a recessive disorder which is characterized by cerebellar ataxia, telangiectases, and a predisposition to malignancy.^37^ As only one *ATM* variant was found in the patient, this will not be the sole cause of the progressive movement disorder. However, it cannot be excluded that this variant has an influence on the course of the patient's disease. As heterozygous carriers of *ATM* variants may have an increased risk of cancer,^38^ this variant could be an additional genetic cause for the development of the patient's hepatocellular carcinoma.

After analysis of the exome data, only the pathogenic variant c.455G>A (p.D152G) in *KCND3* can be linked to the observed ataxic movement disorder. The father and sister of the index patient showed similar symptoms, suggesting an autosomal dominant inheritance of the movement disorder consistent with SCA19/22.^5^ The index patient also had dysarthria and dysphagia, mild intention tremor and reduced reflexes. Cognitive function was preserved at the last examination. There was no cardiac involvement. Similar signs and symptoms have been described in patients with SCA19/22.^12^

The variant c.455G>A leads to the aa change p.D152G, which replaces the acidic aspartate with a neutral glycine in the intracellular N‐terminus of the potassium channel Kv4.3. At the same codon, base changes were reported that result in an aa change from aspartic acid to asparagine (c.454G>A; p.D152N; rs1183337083), to histidine (c.454G>C; p.D152H), or to glutamic acid (c.456C>G; p.D152E). These variants were each found once in 1,461,862 alleles (gnomAD gene browser, MAF = 0.0000007). These variants have not yet been associated with a disease and there is no other evidence in the literature. Following functional analysis of p.D152G, the variant was reclassified as pathogenic (PS3, PM1, PM2, PP2, PP3, and PP4).

To date, pathogenic missense variants of *KCND3* that lead to SCA19/22 were located in the transmembrane domains, the pore loop or the C‐terminus of the derived protein Kv4.3.^5^, ^6^, ^7^, ^8^, ^9^, ^10^, ^11^, ^12^, ^13^, ^14^, ^15^, ^16^, ^17^ In the functionally closely related potassium channel Kv3.3, the variant p.D129N, which is also localized in the N‐terminus, is associated with SCA13.^39^ Similar to SCA19/22 caused by Kv4.3 alterations, pathogenic variants in Kv3.3 (SCA13) cluster in the transmembrane domains and the C‐terminus. Therefore, pathogenic variants near the N‐terminus appear to be less common, but still affect channel activity.

The N‐terminus of Kv4.3 harbours the T1 domain, which is important for tetramerization of channel monomers and binding of β‐subunits. T1 is conserved in potassium channels Kv1‐4.^40^ In Kv4.3, T1 includes aa 40–148 and it was shown that the alteration of aa residues of T1 results in limited tetramerization and rapid degradation of channel monomers.^41^ In Kv1.4, acidic aa residues of the T1‐S1 linker are important for the channel inactivation process.^42^ It can be assumed that aa 152 of Kv4.3 is important for tetramerization and KChIP2b binding due to its spatial proximity to the T1 domain and thus influences the channel properties, as we have shown in electrophysiological studies.

Variant p.D152G reduces the current of the Kv4.3 channel by at least 50%. This reduction is similar to that of other pathogenic variants associated with SCA19/22,^5^, ^6^, ^7^, ^14^ although these measurements are done with supplemented KChIP2b. These results are even more surprising because the previously published Kv4.3 variants associated with a neurological phenotype are exclusively located in regions expected to have an immediate influence on permeation or gating, such as transmembrane domains, the pore loop or the C‐terminus.

With respect to channel voltage dependence, the variant p.D152G results in minor differences to the Kv4.3 WT channel. Therefore, the pathogenic effect is most likely caused by the current reduction.

Co‐expressed WT and variant Kv4.3 channels are predicted to form a mixed heteromeric population. The current decrease in these heterocomplexes is even more pronounced than in the homomeric variant channels. This strong effect on WT/p.D152G Kv4.3 channels suggests that the variant affects the Kv4.3 channel in a dominant‐negative manner, meaning that the function of the WT subunits is impaired in the presence of variant subunits. This phenomenon was also reported for Kv4.3,^6^, ^11^, ^13^ and is well known for multimeric potassium channels and other ion channels.^43^

In heart and brain, Kv4.3 forms heterooctamers with KChIP2b.^21^ KChIPs stabilize and influence the inactivation behaviour of Kv4 channels. When co‐expressed with KChIP2b, the current amplitude of p.D152G is similar to that of the WT channel. It is possible that the β‐subunit supports tetramerization of variant channels by stabilizing the complex. In terms of inactivation behaviour, WT/p.D152G channels are inactivated at a more negative potential than WT channels. Co‐expression of KChIP2b abolishes this difference. In accordance with other publications, the channels inactivate at less negative potentials when co‐expressed with KChIP2b.^44^, ^45^

Overexpression of Kv4.3 in CHO cells can lead to cytoplasmic aggregates, likely localized in the endoplasmic reticulum (ER).^11^ This was also seen with co‐expressed KChIP2b.^11^ Another group found impaired trafficking of variant channels to the plasma membrane due to ER retention. Membrane localization of variant tetramers and WT/ variant heterocomplexes was rescued by co‐expression of KChIP2b.^6^, ^46^ Based on the results of imaging in living cells, it seems possible that delayed transport of the variant channels to the cell membrane or problems with the incorporation of the tetramers into the membrane lead to a reduction in ionic current and thus contribute to the phenotype of the patient.

Overall, Kv4.3 p.D152G affects channel activity, possibly by destabilizing the complex and impairing channel transport to the membrane, but does not affect channel gating, consistent with its position close to the T1 domain.

Whole‐exome and copy‐number analysis was critical in elucidating the complex phenotype of the index patient. The p.D152G variant in Kv4.3 appears to be responsible for the ataxic movement disorder observed in the family. Functional studies have shown that despite its localization in the N‐terminus, the new variant has a substantial effect on Kv4.3 channel function, comparable to that described for variants in transmembrane domains. The effects of p.D152G on channel function, and in particular on trafficking, are relatively subtle and may depend on the cellular context, which could explain the exclusively neuronal phenotype without obvious cardiac defects. This may also explain the relatively small effects observed in the heterologous expression system, which may not closely mimic the neuronal environment of cerebellar neurons.

To our knowledge, this is the first time that a pathogenic Kv4.3 N‐terminus variant leading to SCA19/22 has been identified and characterized.

## AUTHOR CONTRIBUTIONS

**Marlen Colleen Reis:** Data curation (equal); formal analysis (equal); investigation (equal); methodology (equal); validation (equal); visualization (equal); writing – original draft (lead); writing – review and editing (equal). **Laura Mandler:** Investigation (equal); methodology (supporting); validation (supporting); writing – review and editing (supporting). **Jun‐Suk Kang:** Investigation (supporting); methodology (supporting); resources (supporting); writing – review and editing (supporting). **Dominik Oliver:** Formal analysis (supporting); methodology (supporting); resources (equal); supervision (equal); validation (equal); writing – review and editing (equal). **Christian Halaszovich:** Formal analysis (equal); investigation (equal); methodology (equal); resources (supporting); software (equal); supervision (equal); validation (equal); writing – review and editing (equal). **Dagmar Nolte:** Conceptualization (lead); formal analysis (equal); project administration (lead); resources (equal); software (equal); supervision (equal); visualization (supporting); writing – original draft (supporting); writing – review and editing (equal).

## FUNDING INFORMATION

No relevant funding was received.

## CONFLICT OF INTEREST STATEMENT

The authors have no conflict of interest.

## CONSENT

The patient signed a consent form for their data to be published.

## Supporting information


Data S1.

## Data Availability

Data generated during this study are included in this manuscript and in supplementary files. Material (plasmids) are available on request from the authors.
