# Supplementary material for: A novel KCND3 variant in the N‐terminus impairs the ionic current of Kv4.3 and is associated with SCA19/22
Source: J Cell Mol Med. 2024 Aug 24;28(16):e70039. doi: 10.1111/jcmm.70039 (PMC11344468; doi:10.1111/jcmm.70039)
Supplement: Supplementary file 1 — Data S1. [file JCMM-28-e70039-s001.pdf]

Supplementary Information

Journal of Cellular and Molecular Medicine

A novel KCND3 variant in the N-terminus impairs the ionic current of Kv4.3 and is associated with SCA19/22

Marlen Colleen Reis[1], Laura Mandler[1], Jun-Suk Kang[2], Dominik Oliver[3], Christian Halaszovich[3*], Dagmar Nolte[1*]

1 Institute of Human Genetics, Justus-Liebig-University Giessen, Germany

2 Department of Neurology, Goethe-University Frankfurt, Germany

3 Institute of Physiology, Philipps-University Marburg, Germany

* Contributed equally to last authorship

Present Address:

Laura Mandler

Department of Neurology, Justus-Liebig-University Giessen, Germany

Jun-Suk Kang

Neuropraxis, Frankfurt, Germany

Corresponding author:

Dagmar Nolte, Institute of Human Genetics, Justus-Liebig-University Giessen

Schlangenzahl 14, 35392 Giessen, Germany.

Tel.: +49-641-9941615, FAX: +49-641-9941609

dagmar.nolte@humangenetik.med.uni-giessen.de

ORCID: 0000-0001-5710-8790

Supplementary Information 1:

Table S1A: Primer sequences for *KCND3* sequencing

Ex1 F	5´-TCACTCACCAGTGATCCTTTCC-3´
Ex1 R	5´-GACTCCCTCCTCCTCTACCCA-3´
Ex2 F	5´-AACAGGTGAATGATTGGCAG-3´
Ex2 R	5´-CCTGTGAGGAGCTCTAGTCC-3´
Ex3 F	5´-AGCCAGCCTCACAGCTTCA-3´
Ex4R	5´-GCCCAGAGTGAAGATGTGAGT-3´
Ex5F	5´-TCCAGAACAGAGACAGGCAG-3´
Ex5R	5´-GAGCGGCTAGAGGATCCT-3´
Ex6F	5´-TCCTCCTAGTTACCACGAGCA-3´
Ex6R	5´-GCTAGCAGCGTGAACCTCAG-3´
Ex6.001F	5´-GCAGAATGCTGGGAATGAG-3´
Ex6.001R	5´-GAATCAGCAGCACATGCAC-3´

Supplementary Text S1B: Panel of ataxia genes

ABCB7, ABHD12, AFG3L2, ANO10, APTX, ARSA, ATCAY, ATM, ATP1A3, ATP8A2, ATXN1, ATXN10, ATXN2, ATXN3, ATXN7, CA8, CACNA1A, CACNA1G, CACNB4, CAMTA1, CAPN1, CCDC88C, CLCN2, COA7, COQ8A, CP, CTBP1, CWF19L1, CYP27A1, DARS2, DDHD2, DNAJC5, DNMT1, EIF2B1, EIF2B2, EIF2B3, EIF2B4, EIF2B5, ELOVL4, ELOVL5, FGF14, FLVCR1, FXN, GBA2, GFAP, GOSR2, GRID2, GRM1, HEXA, HEXB, HSD17B4, ITPR1, KCNA1, KCNC3, KCND3, KCNJ10, KIF1C, MARS2, MRE11, MTTP, NKX6-2, NPC1, NPC2, PDYN, PEX7, PHYH, PLA2G6, PLD3, PMPCA, PNKP, PNPLA6, POLG, POLR3A, PRICKLE1, PRKCG, PRRT2, PUM1, RNF170, RNF216, SACS, SAMD9L, SCN2A, SCYL1, SETX, SIL1, SLC1A3, SLC2A1, SNX14, SOD1, SPG7, SPTBN2, STUB1, SYNE1, TBP, TDP1, TDP2, TGM6, TMEM240, TPP1, TTBK2, TTC19, TTPA, TUBB4A, TWNK, VAMP1, VLDLR, VPS13D, WDR81, WFS1, WWOX, XRCC1

Supplementary Figure 1:

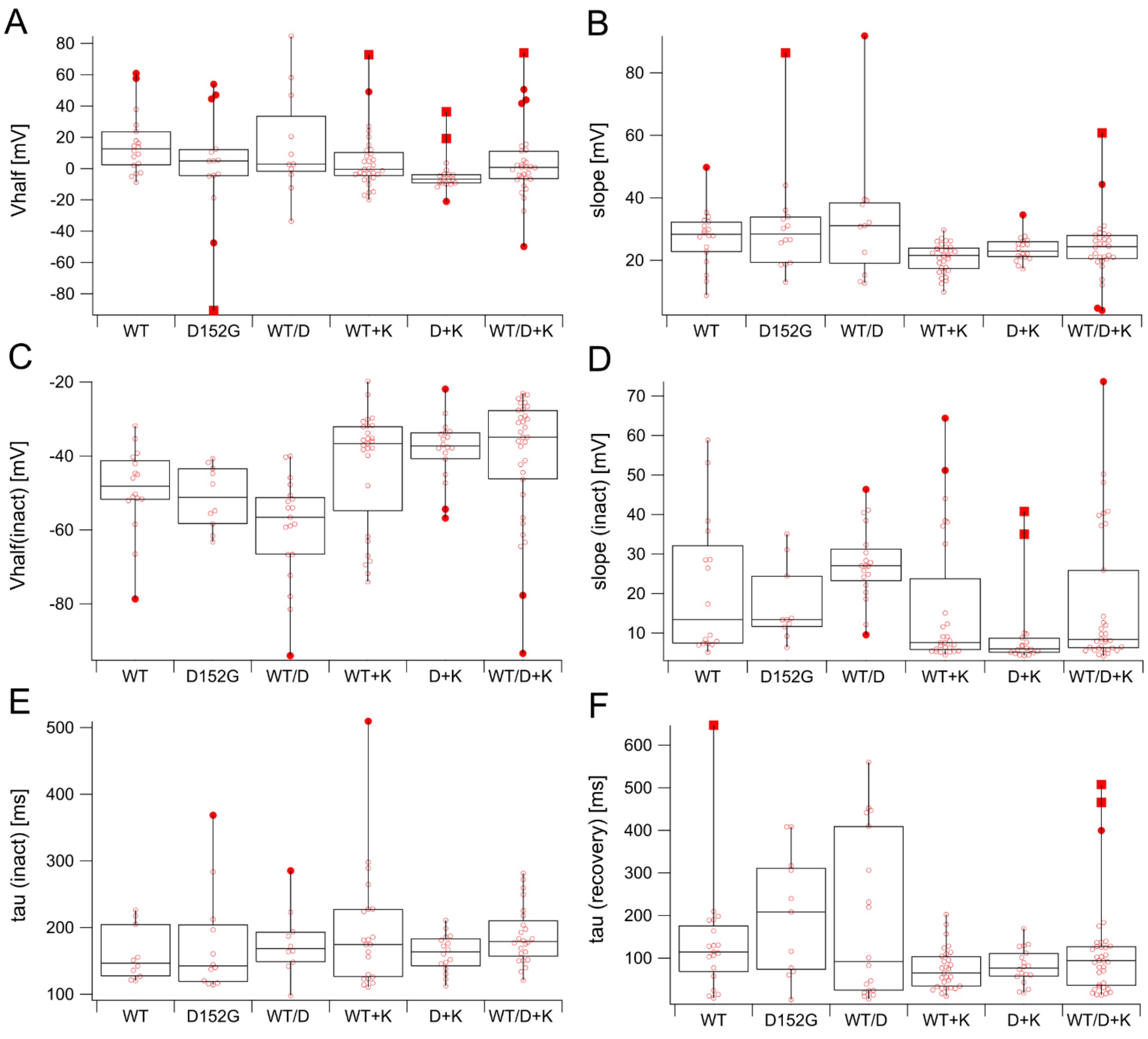

Figure S1 Boxplots of activation and inactivation parameters of Kv4.3. Each dot represents one experiment. The box includes the interquartile range. Filled dots represent outliers, filled squares are far outliers. D= D152G; K= KChIP2b. (A) Vhalf, (B) slope-factor, (C) Vhalf(inact), (D) slope-factor (inact), time constant tau of (E) inactivation and (F) recovery from inactivation

Supplementary Figure 2:

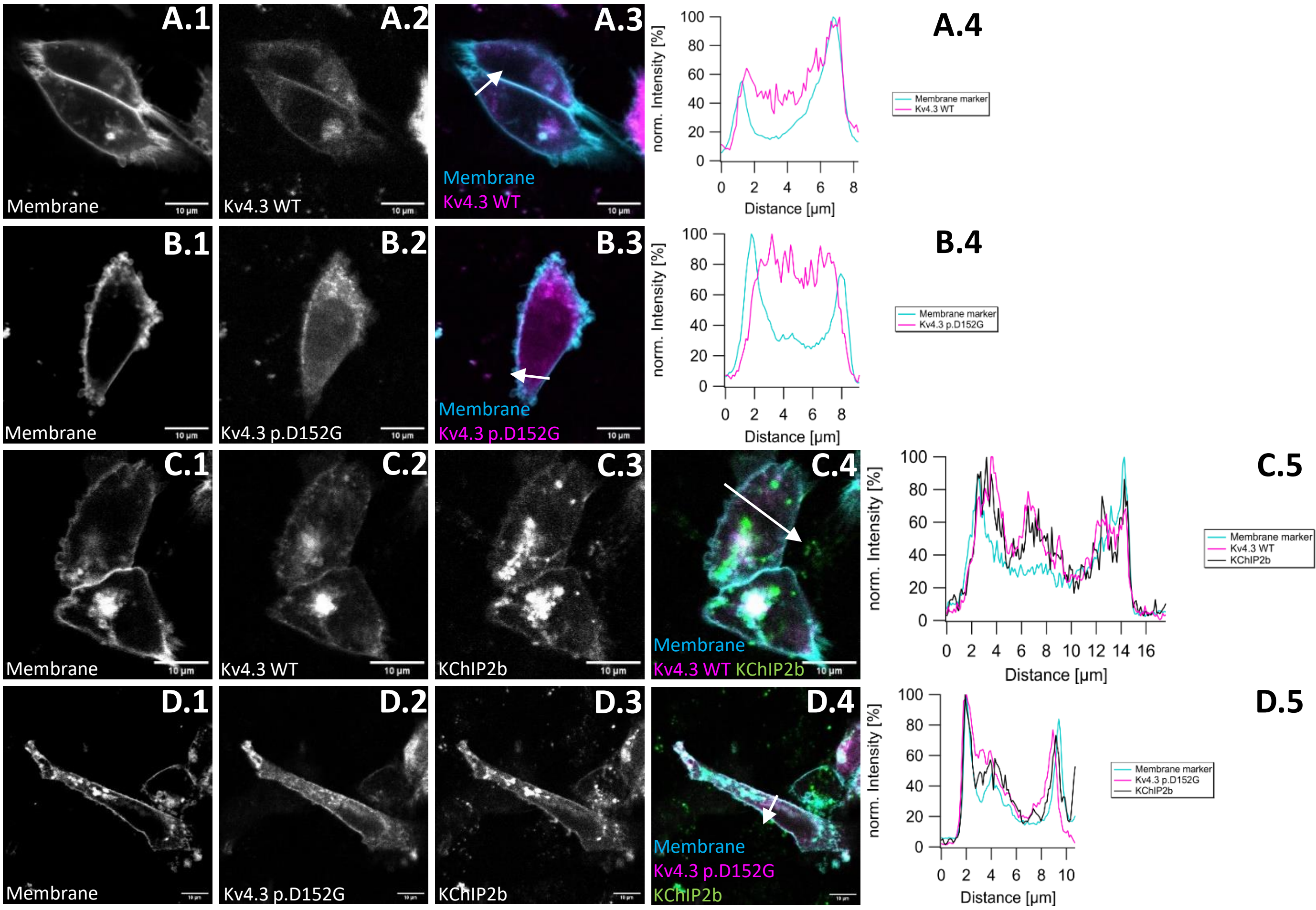

Figure S2 Confocal live-cell imaging of transfected CHO cells. Individual channels are shown in white. (A) (A.1) Membrane marker Lyn11-mCFP was co-transfected with (A.2) Kv4.3 WT channel. (A.3) The overlapping regions of the membrane marker (cyan) and Kv4.3 (magenta) are shown in white. Fluorescence intensity profiles were generated along the white arrow. (A.4) Fluorescence intensities of both channels were normalized to 100% and plotted against the distance relative to the starting point of the arrow. The outer intensity peaks of the membrane marker define the cell edge. (B) Co-transfection was also performed with (B.2) Kv4.3 p.D152G. (B.3, B.4) Fluorescence intensity profiles of membrane marker and p.D152G were plotted. (C) (C.1) The membrane marker was co-transfected with (C.2) Kv4.3 WT and (C.3) KChIP2b. (C.4, C.5) Fluorescence intensity profile was plotted along the white arrow against the distance relative to the starting point of the arrow. (D) Co-transfection of the (D.1) membrane marker, (D.2) Kv4.3 p.D152G and (D.3) KChIP2b shows (D.4, D.5) the distribution of variant channel with KChIP2b in the cell membrane and cytoplasm
